# Influence of Harvest Season and Cultivar on the Variation of Phenolic Compounds Composition and Antioxidant Properties in *Vaccinium ashei* Leaves

**DOI:** 10.3390/molecules22101603

**Published:** 2017-09-30

**Authors:** Verciane Schneider Cezarotto, Sandro Rogério Giacomelli, Maria Helena Vendruscolo, Angélica Signor Vestena, Caroll Schneider Cezarotto, Ritiel Corrêa da Cruz, Luana Haselein Maurer, Luana Mota Ferreira, Tatiana Emanuelli, Letícia Cruz

**Affiliations:** 1 Departamento de Farmácia Industrial, Programa de Pós-Graduação em Ciências Farmacêuticas, Universidade Federal de Santa Maria, Santa Maria RS 97105-900, Brazil; verciane@uri.edu.br (V.S.C.); ritieldc@gmail.com (R.C.d.C.); luanamotaferreirra@gmail.com (L.M.F.); 2 Departamento de Ciências da Saúde, Curso de Farmácia, Universidade Regional Integrada do Alto Uruguai e das Missões, Frederico Westphalen RS 98400-000, Brazil; maria.vendruscolo@gmail.com (M.H.V.); angelica_signor@hotmail.com (A.S.V.); k_rollcezarotto@hotmail.com (C.S.C.); 3 Departamento de Ciências Exatas e da Terra, Curso de Química, Universidade Regional Integrada do Alto Uruguai e das Missões, Frederico Westphalen RS 98400-000, Brazil; srgiacomelli@uri.edu.br; 4 Departamento de Tecnologia e Ciência dos Alimentos, Programa de Pós-Graduação em Ciência e Tecnologia dos Alimentos, Centro de Ciências Rurais, Universidade Federal de Santa Maria, Santa Maria RS 97105-900, Brazil; luanahmaurer@gmail.com (L.H.M.); tatiemanuelli@gmail.com (T.E.)

**Keywords:** harvest season, cultivars, phenolics, antioxidant properties, blueberry leaves, *Vaccinium ashei*

## Abstract

The effect of variation of harvest season and cultivar on the total phenolic content (TPC), total flavonoid content (TFC), HPLC-UV/DAD profile and antioxidant properties in *Vaccinium ashei* (Rabbiteye blueberry) leaves grown in Brazil was evaluated. The cultivars collected in December and March were Aliceblue, Powderblue, Climax, Bluegem and FloridaM. It was observed that leaves from March had the highest TPC values (222 ± 1 mg gallic acid equivalents/g to Aliceblue cultivar) and highest TFC values (49.8 ± 0.8 and 48.7 ± 0.7 µg rutin/g to Clímax and Powderblue cultivars, respectively). The chromatographic profile was quantitatively similar, however, the proportions of each compound were influenced by cultivar and harvest season. Chlorogenic acid and rutin were the main identified phenolic compounds, but chlorogenic acid was the most abundant in both harvest seasons. Antioxidant capacities values ranged from 5.80 ± 0.04 to 105 ± 2 µg/mL (DPPH) and 178 ± 5 to 431 ± 8 mmol Trolox/100 g (ORAC). The cultivar Bluegem by March had the highest values in both assays. The results indicate that the blueberry leaves from different cultivars and harvest seasons have different phenolic compounds content and different antioxidant capacities. In addition, the antioxidant properties demonstrated a high correlation with rutin content.

## 1. Introduction

*Vaccinium* genus (blueberry, family Ericaceae), is known around the world due to its several beneficial effects for human health due to its potential activities against a wide range of degenerative diseases such as inflammatory reactions, oxidative stress induced by aging and cardiovascular problems [1,2,3,4]. Commercial interest is mainly focused on the blueberry fruits, which are consumed fresh or processed, and the blueberry leaves are considered a waste or byproduct which so far were discarded, what could be a mistake [5,6,7,8]. As the total phenolic compound concentration in leaves is three times higher than those observed in the fruits [9,10,11,12] there is a growing scientific interest in them.

Berry phenolics comprise a wide variety of secondary metabolites divided into phenolic acids (such as hydroxybenzoic and hydroxycinnamic acids), flavonoids (flavonol, flavanol and anthocyanin) and condensed (proanthocyanidin) and hydrolysable tannins [12]. Regarding these compounds, several studies have demonstrated numerous benefits related to their high consumption, for example, to prevent oxidative damage originated by reactive oxygen species [13,14,15].

However, the phenolic contents and their related pharmacological activities are often dependent on pre- and post-harvest factors such as species (intraspecies and interspecies differences), environmental characteristics (climatic conditions, humidity and brightness), agronomic features (soil, water supply, use of fertilizers or manure), ripeness, harvesting, transportation method, storage, drying process and extraction methods [7,15,16,17,18]. Hence, the plant chemical composition is influenced by the seasons and may present variability through the months, leading to different final bioproducts with variable composition and pharmacological properties [17]. Due to this, it is essential to evaluate the optimal cultivation time for the highest amount of active compounds and maximum biological activity [19].

Many reports have suggested an antioxidant potential of blueberry leaves [6,9,10,20], but there is no studies about how the harvest season or cultivars influence the activity of blueberry leaves [4,5,7,8,15]. Further, to the best of our knowledge, no reports were found about the variability of phenolic compounds in the blueberry leaves regarding the harvest season for rabbiteye blueberry growing in Brazil. The rabitteye blueberry group is the main cultivar in Brazil because of its tolerance to heat, low demand in the cold season, early flowering and prolonged period between flowering and maturation [21].

Therefore, considering the variations of phenolic composition and consequently the biological activity differences among the different cultivars, the harvest period and the limited information on the phenolic composition of rabbiteye blueberry leaves produced in Brazil, the main objectives of this study were: (1) to evaluate the total phenolic and flavonoids content of blueberry leaves of five different varieties (Clímax, Bluegem, Aliceblue, Powderblue and FloridaM) collected during two different harvest times: December and March; (2) to assess in details the metabolite profile of the phenolic compounds by HPLC-UV/DAD; (3) to determine the antioxidant properties in terms of DPPH and ORAC; and (4) to correlate the variation of the phenolic composition and antioxidant properties with the different times of harvest and the blueberry leaves variety. The results in this study could help decide which is the best cultivar and harvest period to benefit from the maximum pharmacological effects of the blueberry leaves.

## 2. Results and Discussion

### 2.1. Influence of the Cultivar and Harvest Season on Phenolic Compounds Contents

The TPC and TFC quantification in rabbiteye blueberry leaves from different harvest seasons and cultivars is shown in Table 1. The leaves collected in March presented the highest TPC (ranging from 154 ± 1 to 222 ± 1 mg GAE/g) and TFC contents (ranged to 49.8 ± 0.8 to 38.3 ± 0.8 µg rutin/g) (Table 1) confirming that the leaves’ phytochemical composition varies according to the stages of the plant growth [15] and the plant maturity status is reflected the physiological, biochemical and structural processes of the plant tissue [5].

There are many reports about TPC and TFC of blueberry leaves and fruits, what makes it possible to notice that the *Vaccinium* genotype and the harvest season are factors that exert a great influence on the content of these antioxidant compounds. Zhu et al., observed that for the aqueous extract of blueberry leaves (*Vaccinium ashei*) cultivated in China, in different seasons, the highest TFC (114.21 ± 0.03 mg rutin equivalent/g extract) was reported in May and the highest TPC (425.2 ± 0.2 mg gallic acid equivalent/g extract) in November [8]. Similarly, Li et al., reported that rabbiteye blueberry (*Vaccinium ashei*; cv. Brightwell) from Nanjing (China), harvested in July had the highest TPC (339 ± 3 mg GAE/g DW) and TFC (198 ± 2 mg of quercetin/g DW) in the extract of leaves in comparison with fruits and pomace [6]. Routray and Orsat observed that highbush blueberry (*Vaccinium corymbosum*) leaves had a high amount of total phenolics in October (for Nelson 152 ± 3 and for Elliot 156 ± 2 mg GAE/g dry matter) [15].

According to Venskutonis and co-workers, during seasonal development, blueberry plants first concentrate the secondary metabolites in the fruits, but later those metabolites are concentrated in the leaves vegetative portion [7]. This observation corroborates the results obtained in this study. On the other hand, these data are not in agreement with Percival and Mackenzie’s report. In that study during harvest, blueberry (*Vaccinium angustifolium*) leaves presented a higher total phenolic content in green leaf tissues at harvest than those observed two weeks after the collection period [22].

In Brazil, the flowering of blueberry fruits starts in August and ends between early September and the end of October. The blueberry fruit maturation starts during the second half of December until January and the harvest period lasts 37 days [21]. Leaves from December (late spring and early summer) are from the end of fruit ripening and leaves from March (late summer and early autumn) correspond to the phenological stage of the plants when pruning occurs. 

Considering the effect of cultivars on TPC and TFC values, the Clímax and Aliceblue cultivars showed higher TPC levels for both collections (133.6 ± 0.4 to 222 ± 1 mg GAE/g). Regarding the TFC quantification, the Clímax from December showed (32.3 ± 0.2 µg/g) and Bluegem (45.2 ± 0.5 µg/g), Powderblue (48.7 ± 0.7 µg/g). The Clímax (49.8 ± 0.8 µg/g) from March presented the highest compound contents. Ehlenfeldt and Prior previously reported TPC values from fruits and leaves of 87 *Vaccinium corymbosum* cultivars from late July [9]. In the leaves, phenolics values were higher than in the fruit, ranging from 23.6 GAE/g of fresh weight (cv. Reka) to 77.4 GAE/g of fresh weight (cv. Little Giant), with a mean of 44.80 GAE/g of fresh weight. In dried blueberry leaves (*Vaccinium corymbosum*; cv. Bluecrop), collected at the beginning of August, Skupien et al., reported a TPC of 111.5 mg% [23]. 

### 2.2. Phenolic Compounds Identification by HPLC

**Figure 1:**
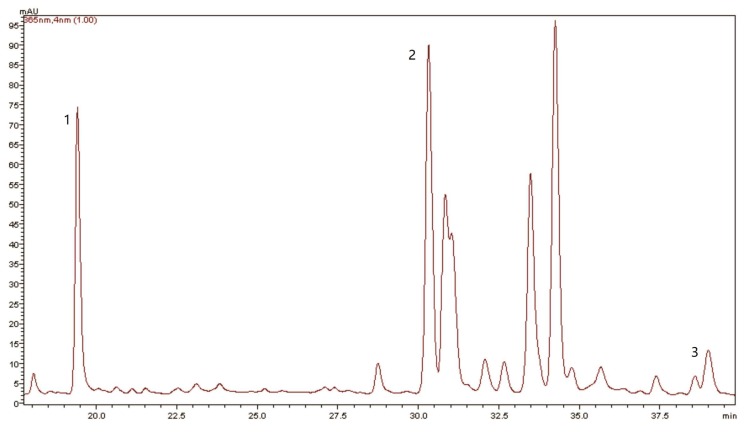
Typical HPLC chromatogram of rabbiteye blueberry leaves, injected volume 40 µL, at λ = 365 nm. Peaks: 1: chlorogenic acid; 2: rutin; 3: quercetin

**Figure 2:**
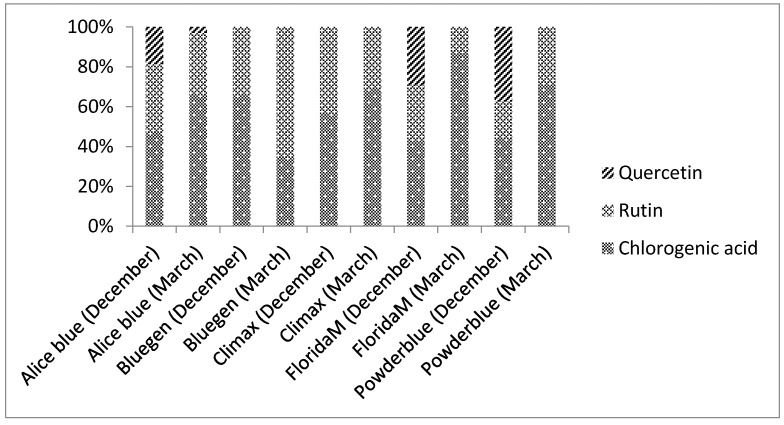
Percentages of the phenolic compounds chlorogenic acid, rutin and quercetin of rabbiteye blueberry leaves from different harvesting months and cultivars by HPLC-UV/DAD.

A typical HPLC chromatogram of solution and percentages of phenolic compounds in blueberry leaves extracts from different harvest months and cultivars are shown in Figure 1 and Figure 2, respectively. All blueberry leaves extracts had similar phenolic composition, however quantitative differences were observed depending on the cultivar and collection month (Table 2).

Three phenolic compounds were identified: chlorogenic acid (retention time - tR = 19.3 min, peak 1), rutin (tR = 30 min, peak 2) and quercetin (tR = 38 min, peak 3), comparing the retention time and UV spectra with 11 commercial standards (Figure 1). The quantification of chlorogenic acid, rutin and quercetin by HPLC-UV/DAD was based on reference standard calibration curves. Calibration curve for chlorogenic acid: y = 168508x − 52225 (r = 0.9980); rutin: y = 72823x − 1900.7 (r = 0.9982); and quercetin: y = 187893x − 163671 (r = 0.9984).

Chlorogenic acids (3-*O*-caffeoylquinic acids) were the most prevalent phenolic compound in blueberry leaf extracts (2.03 ± 0.03 to 21.28 ± 0.05 mg/g DW; corresponding to 32.2 and 87%, respectively) (Table 2 and Figure 2), which was consistent with Ferlemi et al. [12] who reported that the leaves are one the richest sources of chlorogenic acids and previous leaves analysis from 38 rabbiteye blueberry, 37 northern highbush blueberry and 29 southern highbush blueberry leaves collected in China in October showed substantial differences from each other. The eight chlorogenic acids were detected and this compound was the most abundant phenolic compounds in leaves of all cultivars [4].

In present study, the content of rutin ranged from 2.59 ± 0.04 to 15.8 ± 0.1 mg/g DW (corresponding to 13 and 64.8%, respectively). Quercetin was not detected in the chromatograms of some cultivars (0.83 ± 0.02 to 11.9 ± 0.2 mg/g DW) 

In a study performed by Ferlemi et al. (2015) *Vaccinium corymbosum* leaf extract (cv. Bluecrop and Patriot) demonstrated by LC-ESI/MS and HPLC-DAD five major polyphenols (chlorogenic acid, rutin, hyperoside, isoquercetin and quercetin aglycone) which were able to protect the affected tissues (cortex, liver, from the overdose of selenite) and enhanced the antioxidant state of the least perturbed tissues [24].

Chlorogenic acid is an ester of caffeic acid and quinic acid, while rutin (quercetin-3-*O*-rutinoside) is a quercetin glycoside. These compounds have a well-established antioxidant activity [25,26]. The chlorogenic acid antioxidant activity is attributed to the catechol phenyl ring and the double bond together with the catechol group serving as a site for the attack of free radicals [27]. Besides, chlorogenic acids have activity against hepatocellular carcinomas and fibroblastic sarcomas, as well as anti-inflammatory, cardioprotective, cardioprotective, and neuroprotective properties [4,12]. In turn, rutin has demonstrated a number of pharmacological properties, including cytoprotective, vasoprotective, anticarcinogenic, neuroprotective and cardioprotective activities [28] suggesting the pharmacological potential of blueberry leaves.

Table 2 shows the effect of harvest season on chlorogenic acid values. It is possible to observe that the Powderblue and Aliceblue cultivars from March showed higher chlorogenic acid content (21.28 ± 0.05 and 18.21 ± 0.05 mg/g of DW, respectively). Besides, FloridaM, Climax and Aliceblue cultivars from December showed higher chlorogenic acid content (17.34 ± 0.05, 15.87 ± 0.03 and 15.58 ± 0.05 mg/g of DW, respectively). The Bluegem cultivar from March showed higher rutin content (15.84 ± 0.13 mg/g of DW). Quercetin was identified in Aliceblue, Powderblue and FloridaM cultivars and the highest concentrations were observed in the December collection.

This variation was also observed by Zhu et al. where the presence of the chlorogenic acid (34 ± 2, 8.8 ± 0.2 and 14.07 ± 0.02 mg/g), caffeic acid (0.5 ± 0.1, 0.09 ± 0.01 and 0.13 + 0.01 mg/g), rutin (7 ± 1, 2.30 ± 0.03 and 2.6 ± 0.4 mg/g), hyperoside (3 ± 1, 1.1 ± 0.2 and 4.8 ± 0.7 mg/g), galuteolin (27 ± 2 ) and quercitrin (4.2 ± 0.4, 1.33 ± 0.03 and 2.10 ± 0.07 mg/g) was detected in aqueous extracts of blueberry leaves from different seasons, specifically for the samples from the months of May, September and November, respectively [8].

Grace and co-workers showed that UV light stimulates the production of foliar chlorogenic acid content in plants [29]. Fully exposed leaves produced higher levels of chlorogenic acid, whereas in shaded leaves it similar chlorogenic acid contents were found between seasons. Besides, one of the abiotic stresses which affects temperate plants is the low temperature, so in autumn, an increase in the content of a range of cryoprotective substances with the purpose of maximize their cold tolerance it can be seen [30]. Considering this, it is possible to suggest that the plant from March used in this study was already accumulating the metabolites to be prepared for the winter. After this period, the rutin, quercetin and chlorogenic acid concentration in the leaves decreases the development of the flowers in spring and resource allocation shifting from defense to reproduction [19].

### 2.3. Influence of Cultivar and Harvest Season on Extracts Antioxidant Activity

The antioxidant properties of the blueberry leaves extracts from different harvest months and cultivars by the DPPH (2,2-diphenyl-2-picrylhydrazyl) free radical capture method and the oxygen radicals removal ability method (ORAC) are shown in Table 3. The DPPH and ORAC methods are common analyses used to evaluate the antioxidant activities of medicinal plants. To the best of our knowledge, no report on the antioxidant activity of rabbiteye blueberry as a result of different harvesting months and cultivars from Brazil exists.

According to the results expressed in Table 3, the values ranged from 5.80 ± 0.04 to 105 ± 2 µg/mL (DPPH) and 178 ± 5 to 431 ± 8 mmol Trolox/100 g (ORAC). In general, the cultivars from March showed higher average antioxidant properties than the ones from the December collection, regardless of the test employed. Concerning the influence of the cultivar type, the Bluegem from March presented the highest antioxidant properties in both assays (5.80 ± 0.04 µg/mL DPPH and 431 ± 8 mmol Trolox/100 g ORAC). 

The results demonstrated that there was a variation in the antioxidant activity depending on the cultivar and the collection period, regardless the method used in the evaluation. As previously exposed, changes in environmental conditions during the different seasons and genetic predisposition can explain these variations [15]. For the species *Vaccinium corymbosum*, Ehlenfeldt and Prior reported values of about 490.4 µmol Trolox/g (ORAC) for hydroalcoholic extracts of leaves from different cultivars [9] and Pervin, Hasnat and Lim reported values of about 0.12 ± 0.003 mg/mL by DPPH assay [20].

Moreover, in studies with blueberry fruits, the total antioxidants activities in six different varieties varied about 2.6 times according to the ORAC assay, and 2 times by the peroxyl radical scavenging capacity (PSC) assay [31]. The same was observed by Cardeñosa and co-workers, where the genotype influenced the antioxidant capacity and the content of the three groups of phenolics in blueberry fruits [32]. In accordance of Sarkar and co-workers, the genotype versus environment interactions are most critical in the in vitro anti-diabetic-relevant functionalities of blueberry bioactives [33].

The correlation between the phenolic compounds (total phenolic, flavonoid content, chlorogenic acid and rutin contents) with antioxidant activity (DPPH and ORAC) of blueberry leaves from December and March is shown in Table 4.

According to Table 4, rabbiteye blueberry leaves from December showed the highest correlation coefficient for interaction between DPPH and chlorogenic acid (0.99), which describes a strong positive correlation (0.8 < r < 1). On the other hand, interactions between DPPH and total phenolics (0.76) and the interaction between ORAC and total flavonoids (0.69) presented a moderate positive correlation (0.5 < r < 0.8). Despite the high overall phenolics and flavonoids total content, no correlation was found for interactions between phenolics, flavonoids total content and chlorogenic acid versus DPPH or ORAC to blueberry leaves from March (Table 4).

In contrast, rabbiteye blueberry leaves from March showed a strong positive correlation (0.8 < r < 1) between rutin and DPPH (0.83) and between rutin and ORAC (0.80). The same was observed between DPPH and chlorogenic acid (0.98) to rabbiteye blueberry leaves from December. This correlation helps to understand the contribution of rutin to the antioxidant capacity of rabbiteye blueberry leaves. In accordance with Yang and co-workers [34], rutin play an important role in terms of antioxidant capacity against numerous in vitro antioxidant systems and this capacity depends on its concentration.

In conclusion, in view of the different phenolic compounds contents and the antioxidant properties identified in the leaves, this study contributes to better understand the influence of different cultivars and harvest seasons, as well as, it extends the blueberry aplications not limiting them only to its fruits. The results demonstrated that the Bluegem variety harvested in March is the most promising, and considering the high cost associated with growing blueberry fruits, the use of their leaves can be considered advantageous in this aspect. The byproducts derived from leaves could be used as infusions being a coadjuvant treatment for many conditions where oxidative stress is involved. Moreover, these byproducts can be viewed as intermediate products to produce final pharmaceutical and nutraceutical dosage forms.

## 3. Materials and Methods 

### 3.1. Rabbiteye Blueberry Leaves

The rabbiteye blueberry leaves samples (*Vaccinium ashei*) were directly collected from the producer at the orchard in Golden Valley in the city of Erechim (Rio Grande do Sul State, Brazil) in December 2013 and March 2014 (coordinates 27°38'3" S and 52°16'26" W). The following cultivars: Alice Blue, Flórida M, Bluegem, Clímax and Powderblue were used. Dried voucher specimens are preserved in the Herbarium of the Department of Botany at the Federal University of Rio Grande do Sul (UFRGS, Porto Alegre, RS, Brazil) under the registeration numbers ICN 186811, ICN 186812, ICN 186813, ICN 186814, ICN 186815, respectively. The extract preparations were carried out with dried leaves obtained under the following conditions: the leaves were dried in an oven (40 °C) and grounded in a knife mill (80 µm).

### 3.2. Reagents and Standards

Only chemicals of analytical grade were used. Methanol, ethanol, ascorbic acid, gallic acid and chlorogenic acid were purchased from Merck (Darmstadt, Germany). Folin-Ciocalteau phenol reagent (2 mol/L), aluminum chloride, sodium carbonate, DPPH radical (1,1-diphenyl-2-picrylhydrazyl), coumarin, 4-hydroxycoumarin, catechin, quercetin, rutin, chrysin, kaempferol and rosmarinic and caffeic acids were acquired from Sigma Aldrich Chemical Co. (St. Louis, MO, USA). Fluorescein (3′,6′-dihydroxyspiro[isobenzofuran-1[3*H*],9′[9*H*]-xanthen]-3-one), Trolox (6-hydroxy-2,5,7,8-tetramethylchroman-2-carboxylic acid) and (AAPH) 2,2′-azobis-2-amidinopropane were obtained from Aldrich (Milwaukee, WI, USA). For the ORAC assessment, a fluorescein stock solution (407 μmol/L) was prepared in a potassium phosphate buffer (75 mmol/L; pH 7.4) and kept at 4 °C in the dark. The work solution of fluorescein (81 nmol/L) was freshly prepared after dilution with phosphate buffer.

### 3.3. Extracts Preparation

A total of 60 g of the powdered material of each cultivars were extracted three times (three aliquots of 400 mL) by maceration using water-ethanol (1:1, *v*/*v*) for 72 h at room temperature [35]. The blueberry hydroethanolic leaf extracts were concentrated under low pressure at 50 °C and dried by lyophilization. The extracts were stored in amber glass at 10 °C to further analysis.

### 3.4. Total Flavonoids Content (TFC)

Total flavonoid contents of different extracts were quantified by the method described by Woisky and co-workers [36] based on the flavonoid-aluminum complex formation. An aliquot of hydroethanolic leaf extract (0.5 mL) diluted in methanol (1 mg/mL) was mixed with aluminum chloride solution (0.5 mL, 2%; *w*/*v*) and methanol (2.5 mL) and incubated for 30 min at room temperature. Absorbance was measured at 420 nm using a UV-Vis spectrophotometer (Perkin Elmer, series 200, North Billerica, MA, USA. This way, the TFC was determined by interpolating the absorbance of the samples against a calibration curve (y = 0.0072 x + 0.0311, r = 0.9983) constructed with rutin standard solution (5 to 150 µg/mL) and expressed as µg of rutin (rutin equivalents) per g of dry weight (DW).

### 3.5. Total Polyphenolic Content (TPC)

The total phenolic content quantification was performed by the Folin-Ciocalteau method [37]. Briefly, Folin-Ciocalteau reagent (0.5 mL; 2 mol/L) was added to hydroethanolic leaf extracts (1 mL) diluted in methanol (0.15 mg/mL) and this mixture was left standing for 5 min before the addition of 20% Na_2_CO_3_ (2 mL). The solution was then resting for 10 min before measurement at 730 nm in the UV-Vis spectrophotometer (Perkin Elmer, series 200). The total phenolic content was expressed in milligrams equivalent of gallic acid (GAE) per g of dry weigh (DW). The equation obtained for the calibration curve of gallic acid in the range of 100–1000 µg/mL was y = 0.0014 x + 0.088 (r = 0.9975).

### 3.6. Polyphenolics Qualitative Identification by HPLC-UV-DAD

High performance liquid chromatography (HPLC-UV-DAD) was performed with a Prominence Auto-Sampler (SIL-20A) equipped with Shimadzu LC-20AT (Shimadzu, Kyoto, Japan) pumps connected to a DGU-20A5 degasser and a CBM-20A integrator. A SPD-M20A UV-Vis DAD and LC Solution 1.22 SP1 software were used. Analyses were carried out using a Phenomenex C_18_ column (4.6 mm × 250 mm) packed with 5 µm diameter particles. Injection volume was 40 µL and the gradient elution was conducted according to the slightly modified method [38]. The UV absorption spectra was recorded in the 200–400 nm range. Each hydroethanolic leave extract was individually screened for the presence of the following polyphenolic compounds: gallic, chlorogenic and caffeic acids, coumarin, 4-hydroxycoumarin, catechin, quercetin, rutin, chrysin, kaempferol and rosmarinic acid. The compound identification was performed by comparing their HPLC retention times and UV absorption spectra with the respective commercial standards. Standard stock methanolic solutions were prepared in the concentration range of 2.5–60.0 μg/mL. Quantification was carried out by integrating the peaks using external standard method at 327 nm wavelength for chlorogenic acids and 365 nm for quercetin and rutin. Chromatographic operations were carried out at room temperature and in triplicate.

### 3.7. Antioxidant Activity Analysis

#### 3.7.1. DPPH Assay

The DPPH assay was used to measure radical scavenging activity [39]. Different levels (250, 125, 50, 25, 10 and 5 µg/mL) of samples were prepared in ethanol. The DPPH ethanol solution (1 mL; 0.3 mM) was added to 2.5 mL of sample solutions at room temperature. After 30 min of incubation, the decrease in absorbance at 518 nm was evaluated. A blank solution was prepared with ethanol (1.0 mL) plus plant extract solution (2.5 mL). As negative control, a DPPH solution (1.0 mL; 0.3 mM) plus ethanol (2.5 mL) was used. The positive controls were the ascorbic acid standard solutions. The inhibition percentage the DPPH solution absorbance was calculated using: 100 – {[(Abssample − Absblank) × 100]/Abscontrol}. The results were expressed as concentration of the extract required to scavenge 50% DPPH free radicals (IC_50_) in µg/mL.

#### 3.7.2. ORAC Assay

The oxygen radical absorbance capacity (ORAC) assay was performed according to Ou, Hampsch-Woodill and Prior with modifications [40]. An aliquot of fluorescein solution (150 μL) was added to diluted extract or Trolox standards (25 μL, 0–96 μmol/L) prepared in phosphate buffer in a black 96-well plate and incubated at 37 °C for 10 min. The reaction was initiated with 25 μL of the peroxyl radical generator AAPH (152 mmol/L) prepared before its use. The fluorescence was measured (λexc = 485 nm and λem = 528 nm) every minute for 90 min using a SpectraMax M5 plate reader (Molecular Devices, Sunnyvale, CA, USA) maintained at 37 °C. Standards and samples were prepared in triplicate. Results for ORAC were determined by graphical regression analysis and expressed as mM of Trolox equivalents per 100 g of extract.

### 3.8. Statistical Analysis

Results were expressed as mean ± standard error mean (SEM). Student’s *t*-test was used for comparison between two means and two -way analysis of variance (ANOVA) followed by student Newmann-Keuls was used for comparison of more than two means. A difference was considered statistically significant when *p* < 0.05. The IC_50_ values were calculated from linear regression analysis. The Pearson correlation analysis between antioxidant activity and total phenolic and flavonoids, chlorogenic acid and rutin content was performed.

## Figures and Tables

**Table 1:** TPC and TFC rabbiteye blueberry leaves from different harvest seasons and cultivars.

Cultivars	TPC (mg/g) ^1^		TFC (µg/g) ^2^	
	December	March	December	March
Bluegem	75.4 ± 0.6 ^a^	170 ± 2 ^a^	18.9 ± 0.2 ^a^	45.2 ± 0.5 ^a^
Powderblue	79 ± 1 ^b^	154 ± 1 ^b^	24.3 ± 0.3 ^b^	48.7 ± 0.7 ^b^
Clímax	133.6 ± 0.4 ^c^	185 ± 1 ^c^	32.3 ± 0.2 ^c^	49.8 ± 0.8 ^b^
FloridaM	93.1 ± 0.5 ^d^	166 ± 1 ^d^	21.3 ± 0.2^d^	38.3 ± 0.8 ^c^
Aliceblue	110 ± 2 ^e^	222 ± 1 ^e^	19.5 ± 0.3 ^a^	39.1 ± 0.6 ^d^
Means ± SD	98 ± 21	179 ± 26	23 ± 5	44 ± 5

Two-way ANOVA followed by Student Newmann-Keuls; Values are expressed as mean ± SEM (*n* = 3). Different letters in the same column indicate significant differences (*p* < 0.05). ^1^ TPC is expressed as milligrams of gallic acid (GAE) per gram of dry weight (DW). ^2^ TFC is expressed as micrograms of rutin per gram of dry weight (DW).

**Table 2:** Phenolic compounds blueberry contents of rabbiteye leaves from different harvesting months and cultivars by HPLC-UV/DAD ^1^.

Cultivars/Compounds	Clímax		Aliceblue		Bluegem		Powderblue		FloridaM	
	December	March	December	March	December	March	December	March	December	March
Chlorogenic acid	15.87 ± 0.03 ^a^	9.8 ± 0.01 ^b^	15.58 ± 0.05 ^a^	18.21 ± 0.05 ^c^	7.41 ± 0.02 ^d^	2.03 ± 0.03 ^e^	8.76 ± 0.01 ^f^	21.28 ± 0.05 ^g^	17.3 ± 0.3 ^h^	14.11 ± 0.07 ^i^
Rutin	12.13 ± 0.02 ^a^	4.38 ± 0.01 ^b^	11.3 ± 0.1 ^c^	8.64 ± 0.09 ^d^	3.73 ± 0.08 ^e^	15.8 ± 0.1 ^f^	3.6 ± 0.1 ^g^	8.9 ± 0.2 ^d^	11.42 ± 0.02 ^c^	2.59 ± 0.04 ^g^
Quercetin	N.D.	N.D.	6.20 ± 0.09 ^a^	0.83 ± 0.02 ^b^	N.D.	N.D.	7.4 + 0.2^c^	N.D.	11.9 ± 0.2 ^d^	N.D.

Two way ANOVA followed by Student Newmann-Keuls; Values are expressed as mean ± SEM (*n* = 3). Different letters in the same line indicate significant differences (*p* < 0.05).^1^ Expressed as milligram per gram of dry weight (DW). N.D.: not detected

**Table 3:** Rabbiteye blueberry leaves antioxidant properties considering different harvesting months and cultivars by DPPH and ORAC methods

Cultivar	IC_50_ for DPPH (µg/mL)		ORAC Values (mmol Trolox/100 g)	
	December	March	December	March
Bluegem	105 ± 2 ^a^	5.80 ± 0.04 ^a^	211 ± 6 ^a^	431 ± 8 ^a^
Powderblue	60.1 ± 0.2 ^b^	12.1 ± 0.6 ^b^	374 ± 2 ^b^	202 ± 9 ^b^
Clímax	25 ± 2 ^c^	12.39 ± 0.02 ^b^	341 ± 5 ^c^	283 ± 6 ^c^
Florida M	25.6 ± 0.1 ^c^	16.0 ± 0.2 ^c^	178 ± 5 ^d^	181 ± 9 ^d^
Aliceblue	24.5 ± 0.2 ^c^	12.8 ± 0.2 ^b^	245 ± 5 ^e^	338 ± 4 ^e^
Means ± SD	48 ± 35	11 ± 4	270 ± 84	287 ± 102.01

Two way ANOVA followed by Student Newmann-Keuls; Values are expressed as mean ± SEM (*n* = 3). Different letters in the same column indicate significant differences (*p* < 0.05).

**Table 4:** Correlation between the total phenolic content (TPC), total flavonoid content (TFC), chlorogenic acid content, rutin content and antioxidant capacities by free radical capture method DPPH (2,2-diphenyl-2-picrilhidrazil) and the ability method removing oxygen radicals (ORAC).

	DPPH		ORAC	
	December	March	December	March
TPC	0.76	0.43 ^a^	0.25	0.40
TFC	0.30	0.08	0.69	0.05
Chlorogenic acid	0.99	0.65 ^a^	0.24 ^a^	0.69 ^a^
Rutin	0.98	0.83	0.21 ^a^	0.80

^a^ showed negative correlation
